# Evaluation of Effect of Honey Sugars Analogue Therapy against Breast Cancer Induced by 1-Methyl-1-nitrosourea in *In Vivo* Breast Cancer Model

**DOI:** 10.1155/2022/6457266

**Published:** 2022-03-27

**Authors:** Sarfraz Ahmed, Sadia Nawaz, Mogana Das Murtey, Muhammad Ibrahim, Waqas Ahmad, Muhammad Asif Idrees, Nur Asyilla Che Jalil, Nor Hayati Othman

**Affiliations:** ^1^Departments of Pathology, School of Medical Sciences, Universiti Sains Malaysia, 16150 Kubang Kerian, Kelantan, Malaysia; ^2^Department of Basic Sciences, University of Veterinary and Animal Sciences Lahore, Narowal Campus, 51600 Narowal, Pakistan; ^3^Institute of Biochemistry & Biotechnology, University of Veterinary and Animal Sciences Lahore, 54000 Lahore, Pakistan; ^4^Basic Sciences and Oral Biology Unit, School of Dental Sciences, Health Campus, Universiti Sains Malaysia, 16150 Kubang Kerian, Kelantan, Malaysia; ^5^Department of Biochemistry, Bahauddin Zakariya University, 60800 Multan, Pakistan; ^6^Department of Clinical Sciences, University of Veterinary and Animal Sciences Lahore, Narowal Campus, 51600 Narowal, Pakistan; ^7^Department of Pathobiology, University of Veterinary and Animal Sciences Lahore, Narowal Campus, 51600 Narowal, Pakistan

## Abstract

The use of honey as a complementary and alternative medicine is associated with vast range of therapeutic promises. It is established that it exhibits potential innumerable medicinal effects which is attributed to it phenolic, flavonoids, and other diverse compounds profile. However, the effect of honey sugars analogue as its major constituent has not been investigated. This study examined the effect of honey sugars analogue (HSA) namely fructose, glucose, maltose, and sucrose in breast cancer-induced albino Sprague–Dawley (SD) rat models. The treatment was administered when first palpable tumour reached 10–12 mm in size by dividing nulliparous rats (*n* = 30) into following groups: Group 0 (negative control, *n* = 10), Group 1 (positive control, *n* = 10), and Group 2 (received 1.0 g/kg body HSA, *n* = 10) over a period of 120 days. The effect of treatment against breast cancer was observed with a slower tumour progression, a lower median tumour size, multiplicity, and weight (*p* < 0.05). The anticancer effect was through amelioration of tumour growth, tumour grading, and haematological parameters. Data also show that HSA administration induces an increased susceptibility of expression of proapoptotic proteins such as Apaf-1, caspase-9, IFN-*γ*, IFNGR1, and p53, and a reduced expression of antiapoptotic proteins such as E2, ESR1, TNF-*α*, COX-2, and Bcl-xL 1 in their mechanisms of action. HSA behaves akin to honey. Thus, HSA may modulate breast cancer as an analogue or major profile of honey.

## 1. Introduction

Cancer has been identified in about 11 million people and is responsible for 7.6 million deaths globally per year [1]. Generally, cancer is an abnormal growth of cells. It starts as an onset from a single transformed cell. Its genesis is characterized by the swift proliferation, invasion, and metastasis [2]. This dynamic process is activated by various carcinogens, tumour promoters, and inflammatory agents. The whole modulation is controlled through transcription factors, proapoptotic proteins, antiapoptotic proteins, protein kinases, cell cycle proteins, cell adhesion molecules, cyclooxygenase-2 (COX-2), and other molecular targets [3]. Among different types of cancer, breast cancer is emerging as a rapidly spreading phenotype affecting women in both developed and developing countries. It is recognized as the second most common cancer after lung cancer, the fifth most common cause of death through cancer, and the leading cause of death in women worldwide [4, 5].

Honey, a supersaturated natural product has been recognized as a potential medicinal agent. It has been shown to exert potential antimicrobial, anti-inflammatory, anticancer, antiangiogenic, anti-metastatic, immune-stimulant, antiulcer, vasodilative, hypotensive, antihypercholesterolemic, antibrowning, disinfectant, and wound healing effects [6]. It is primarily comprised of sugars such as fructose, maltose, and glucose. Besides sugars, honey constitutes several components such as flavonoids, phenolic acids, carotenoids, amino acids, proteins, enzymes, minerals, vitamins, organic acids, and a group of miscellaneous compounds [7, 8].

The estimated percentage composition of HSA or common sugars in honey has been described as follows: fructose (30–35%), glucose (30–35%), maltose (7–10%), and sucrose (1–3%) [8–10]. The major components of honey, i.e., sugar, particularly fructose and glucose, have also been demonstrated to inhibit the yield of mutagenic activity in different models [11]. Fructose as one of the honey sugars analogue induces apoptosis in malignant hepatocytes with no cytotoxic effects [12]. Earlier studies have shown that fructose and glucose possess either carcinogenic and anticarcinogenic or mutagenic and antimutagenic properties [13–16]. Though the phenolic, flavonoids and other constituents of honey have been well studied, so far none has taken the sugars part of honey into cognizance for its possible biological effects. Honey sugars analogue is far opposite to table sugar which is sucrose only, while honey sugars include fructose, glucose, and maltose as a major constituent.

Our previous studies [17] have established that honey exhibits antibreast cancer effects. This study was conducted to probe the potential effect of HSA, whether honey sugars analogue exhibits anticancer effect or not in breast cancer SD rats model. The potential “therapeutic” effects of HSA in our study were to investigate the antitumoural effects, the histological features, and the tumour grades evaluation. This study also pinpoints a potential therapeutic role of HSA to modulate haematological parameters and the expression of Apaf-1, caspase-9, IFN-*γ*, IFNGR1, p53, E2, ESR1, TNF-*α*, COX-2, and Bcl-xL. We believe this is the first study to report the antibreast cancer activity of sugar part of honey (HSA).

## 2. Materials and Methods

### 2.1. Animals

The experimental procedure used in this study was approved by the Animal Ethics Committee of University Sains Malaysia, Malaysia (USM/Animal Ethics Approval/2011/ (68) (306)). Sprague–Dawley (SD) female rats aged between 28 and 33 days were taken from Animal Research and Service Centre (ARASC), University Sains Malaysia (USM).

### 2.2. Preparation of Honey Sugars Analogue (HSA)

The estimated percentage composition of sugars in honey has been described as follows: fructose 30–35%, glucose 30–35%, maltose 7–10%, and sucrose 1–3% (O. O. Erejuwa et al., 2011; Shin & Ustunol, 2005). Honey sugars analogue was prepared using mentioned percentage of sugars: fructose, glucose, maltose, and sucrose (Merck, Germany) with water in the ratio of 1 : 1 : 0.25 : 0.03, respectively.

### 2.3. Tumour Induction

For tumour induction, the carcinogen MNU (catalog no. N1517-1G, Sigma, USA) was dissolved in 0.9% NaCl solution acidified to pH 5.0 with 0.05% acetic acid by gentle heating up with hot tap water with vigorous shaking [18, 19]. MNU was injected intraperitoneally as per 80 mg/kg body weight of the rats at the age of 40 days.

### 2.4. Treatment Plan

A total of 30 female SD rats were divided into 3 groups with 10 animals in each group. These rats were housed in a standard cage with commercial pine chip bedding in a well-ventilated animal room with a 12 h day/night cycle, maintained on standard and balanced rat feed diet and had free access to water and libitum. The rats were acclimatized to the animal room conditions for at least one week prior to the experimentation. Treatment was started by oral feeding when first palpable tumour reached 10–12 mm in size till day 120. The grouping of the rats was as follows:

- Group 0: negative control; no tumour induction and no honey treatment (normal rats)
- Group 1: positive control; rats bearing breast cancer but no HSA treatment
- Group 2: breast cancer bearing rats treated with HSA 1.0 g/kg body weight/day

The rat breast tissue areas were palpated twice weekly to detect the appearance of cancer masses and to monitor their progression. The number, size, and positions of the tumours were recorded. Tumours were measured in length and width weekly to calculate the size and reduction in size of primary tumours after treatment as described by Tran-Thanh et al. [20]; tumour size = 1/2 (length × width^2^).

### 2.5. Determination of Body Weights

The total body weight of rats was measured using a digital analytical balance (Sartorius AG, Germany) weekly from start of treatment till day terminated. The percentage body weight changes or percentage weight gain were calculated at the end of study (week 16). The actual body weight was calculated by subtracting the weight of tumours at week 16. The percentage actual body changes were also calculated. The formula used to calculate percentage weight gain is described as follows:

  Percentage body weight change or gain (BW change %) = [(FBW − IBW) × 100]/IBW
  Actual body weight = body weight at week 16 − weight of tumours
  Percentage actual body weight change or gain (ABW change %) = [(ABW − IBW) × 100]/IBW
  BW, body weight; IBW, initial body weight; FBW, final body weight; ABW, actual body weight.

### 2.6. Samples Collection

After 120^th^ day of treatment, all the rats used in the present study were subjected to necropsy after intraperitoneal (i.p) injection of pentobarbital 100 mg/kg body weight. The blood samples were collected into EDTA and plain tubes by cardiac puncture using a 10 ml syringe and 23 G needle. Blood samples in plain tubes were left to clot for 2 hours prior to centrifugation for 15 minutes at 4000 rpm (Eppendorf centrifuge, Germany). The serum samples were collected and stored at −80°C till assayed. Tumor masses were examined in vivo then excised. Each cancer was fixed in 10% neutral buffered formalin for histological and immunohistochemical analysis.

### 2.7. Determination of Full Blood Count (FBC)

A total of 8-9 samples were run for FBC for each group. FBC was carried out using an automated cell count analyzer (Sysmex KX-21, Japan) by noncyanide haemoglobin analysis. Auto analyzer was capable to run several parameters for each sample such as haemoglobin concentration, packed cell volume, red blood cell, mean cell volume, mean corpuscular haemoglobin concentration, mean corpuscular haemoglobin, platelet, and white blood cell counts. The equipment sampling aspirated 20 *μ*l well mixed blood samples, and the result of analysis was obtained accordingly.

### 2.8. Histopathological Examination of the Breast Cancer Masses

The breast tumours fixed in buffered formalin were processed using an automated closed system of tissue processor (Tissue Tek® VIP, Japan). Briefly, the processing procedure initiated with fixation, followed by dehydration in a series of graded ethanol, clearing in xylene, and completed with paraffin infiltration blocked in paraffin. The tissues were sectioned (3 *μ*m thickness) using microtome (Leica, Germany). The sectioned tissues were mounted on frosted-end glass slides, deparaffinised, and stained with hematoxylin and eosin using the standard method. A total of 65 H&E breast cancer masses were examined and graded, thirty-nine from the positive control group and twenty-six from 1.0 g/kg HSA.

#### 2.8.1. Grading and Histological Features of Cancer Masses

The stained sections were examined under light microscope at 100×, 200×, and 400× magnification using an Olympus BX41 microscope (Olympus Optical Co. Ltd., Tokyo, Japan). The stained sections were examined for grading and histological features by a pathologist who was blind to the treatment and control. The cancer masses were graded as per human cancers grading system using the modified Bloom and Richardson method [21]. Briefly, the grades were based on three morphological features; tubule formation, nuclear pleomorphism, and mitotic count per high power field (hpf). Each feature was given a score between 1 and 3. Tubule or acinar formation that involves majority of the cancer area was scored 1 (˃75%), moderate degree of area (1–75%) was scored 2, and little or none (˂10%) was scored 3. For nuclear pleomorphism, small nuclei with regular uniform cells were scored 1, moderate nuclear size and variation was scored 2, and marked variation was scored 3. Mitotic figures were assessed at 10 high power field (hpf) of periphery and mitotically active part of the cancer. The total count of the mitotic figures per 10 hpf determines the score of either 1, 2, or 3, while the total scores of the features decide grades. Grade 1 was applied for a total score between 3 and 5, grade 2 for score of 6 or 7, and Grade 3 for score of 8 or 9. Different histological patterns or types of cancers examined were determined.

### 2.9. Determination of Apaf-1, IFN-*γ*, TNF-*α*, and E2 at Serum Level

Seven to eight serum samples per treatment and control groups were analyzed to determine the level of Apaf-1, IFN-*γ*, TNF-*α*, and E2 in 50 *μ*l serum using Apaf-1, IFN-*γ*, TNF-*α*, and E2 ELISA kits (catalog no. BG-RAT10190, Novatein Biosciences Inc.; CSB-E04579r, CSB-E11987r, and CSB-E05110r, COSMO BIO Inc, USA, respectively). Standards included serum of known concentrations of Apaf-1, IFN-*γ*, TNF-*α*, and E2 and a serum blank. The ELISA procedure was performed according to the manufacturer's instructions. The results were obtained by calculating the mean absorbance at 450 nm (spectrophotometer, Thermo Fisher Scientific Inc., Waltham, MA, USA) for each of the duplicate standards, controls and samples as stated by the manufacturer. A standard curve was created by plotting with the absorbance value as the dependent variable (*Y*-axis) and concentration as the independent variable (*X*-axis), results in an equation formatted as follows: *y* = *ax*^2^ + *bx* + *c*, with best-fit straight line, where solving for *x* determined the protein concentration of the sample.

### 2.10. Immunohistochemical Analysis for Apaf-1, Caspase-9, p53, FASLG, FADD, IFNGR1, TNF-*α*, COX-2, ESR1, and Bcl-xL in Breast Cancer Masses

A total of 65 cancer tissues, thirty-nine from the positive control group and twenty-six from 1.0 g/kg HSA were immunohistochemically stained for the markers. Apaf-1 with mouse monoclonal anti-Rat Apaf-1 antigen (catalog no. SC-65891, Santa Cruz Biotechnology Inc., USA; diluted at 1 : 100), caspase-9 Rabbit polyclonal anti-rat caspase-9 Antigen (catalog no. GTX73093, GeneTex Inc., USA; diluted at 1 : 25), FASLG with monoclonal mouse anti-rat FASLG Antigen (catalog no. PAB 8018, Abnova Inc., Taiwan; diluted at 1 : 200), FADD with rabbit polyclonal anti-rat FADD Antigen (catalog no. GTX73104, GeneTex Inc., USA; diluted at 1 : 25), p53 with monoclonal mouse anti-rat p53 antigen (catalog no. PAB 1801, Abcam Inc., UK; diluted at 1 : 50), IFNGR1 with rabbit polyclonal anti-rat IFNGR1 antigen (catalog no. GTX60200, GeneTex Inc., USA; diluted at 1 : 200), TNF*-α* with polyclonal rabbit anti-rat TNF*-α* antigen (catalog no. GTX74120, GeneTex Inc., USA; diluted at 1 : 600), ESR1 with polyclonal rabbit anti-rat ESR1 antigen (catalog no. PAB 18170, Abnova Inc., Taiwan; diluted at 1 : 100), COX-2 with polyclonal rabbit anti-rat COX antigen (catalog no. RB-9072-R7, Lab Vision Inc., USA; ready to use), and Bcl-xL with mouse monoclonal anti-rat Bcl-xL antigen (catalog no. MS-1334-P1, Lab Vision Inc., USA; diluted at 1 : 100). A semiquantitative scoring system developed previously [22] was used to assess the expression of proteins mentioned. The positive stained cells were counted in 10 fields by first author confirmed by pathologist (NHO) in a blinded manner. The data were presented as a percentage of positivity.

#### 2.10.1. Immunohsitochemical Staining and Scoring

Tissues sections with 3 *μ*m thickness were sectioned from the formalin-fixed paraffin embedded blocks and mounted on poly-L-lysine slides. The sections were deparaffinised on the 60°C hot plate for 1 hr to attach the tissue to the slide. This was followed by clearing in xylene and rehydrated with descending concentrations of ethanol to distilled water. Subsequently, endogenous peroxidase activity was quenched using 3% H_2_O_2_ in methanol for 10 minutes at room temperature. Antigen retrieval was achieved to the preference of perspective antibodies followed by blocking of nonspecific binding by incubation with Ultra V Block for 5 minutes at room temperature. The sections were incubated with representative antibodies. Subsequently, washing three times with 1X TBS-Tween 20 was performed. Immunoreactivity of respective antibodies was determined by incubating the tissue sections with the commercially available detection kit, Ultra Vision One Large Volume Detection system HRP Polymer (ready to use). These sections were again rinsed with 1X TBS-Tween 20 three times. The sections were stained with freshly prepared DAB solution (substrate + chromogen). The sections were then rinsed with TBS tween buffer and counterstained with hematoxylin solution for 30 second. Followed by rinsing in running tap water, the sections were then dehydrated in a series of ascending ethanol concentrations; 80% ethanol (5 minutes), 95% ethanol (5 minutes), absolute ethanol (5 minutes), and finally followed by xylene (5 minutes). The sections were mounted with Cytoseal XYL mounting medium to be observed.

Expression of all antibodies was assessed using a semiquantitative scoring system developed by Allred et al. [22]. This scoring system is based on proportion score (PS) and an intensity score (IS). The proportion score is an estimation of the proportion of positive cells on the entire slide divided into following criteria: 0 = no cells stained, 1 = less than 1%, 2 = 1% to 10%, 3 = 11% to 33%, 4 = 34% to 67%, and 5 = ˃67%. The intensity score was an estimation of antibody staining intensity divided into following criteria: 0 = none, 1 = weak, 2 = moderate, and 3 = strong. Both scores (PS + IS) were added together to give a final numerical score ranging from 0 to 8. A combined score of above 2 was considered as positive for proteins expression [23]. Scoring was performed in a double blind manner by three independent investigations. The expression was determined in areas of high expression. The proteins expression classification was initially performed by Dr. Sarfraz Ahmed and later confirmed by the main supervisor (Prof. Dr. Nor Hayati Othman), who had a knowledge of classification. Any disagreement was resolved by discussion to obtain a final score. The hotspots of the proteins expression were captured using an image analyzer (Nikon, Japan).

### 2.11. Statistical Analyses

Data were analyzed using IBM SPSS, Statistics version 22. Fisher Exact test was used to analyze the tumour incidence, latency, and grading. Comparisons between mean values of control and treatment groups were analyzed using one-way ANNOVA with post hoc test of Tukey's honest significance differences (Tukey's HSD). A mixed model two way repeated measures ANOVA was conducted to evaluate the effect of treatments on rats body weight gain and tumour measurements. The time main effect and the experimental groups x time interaction effect were tested using the multivariate criterion of Wilk'slamda (Λ). Comparison of the median values between groups was conducted by Kruskal–Wallis H test followed by Benferroni's correction. *p* value <0.05 was considered statistically significant.

## 3. Results

### 3.1. Tumour Multiplicity and % Reduction in Size of Primary Tumours

At the end of study, the rats in positive control which received no honey treatment (Group 1) showed the highest median number of tumours (tumour multiplicity) compared to the groups treated with HSA (Group 2) (*p* > 0.05) (Table 1).

The used strengths of HSA (Group 2) showed a significant % reduction in the size of primary tumours (in first three developed tumours) compared to the non-treated positive control (Group 1). A significant statistical difference was observed between control and treated group (*p* ≤ 0.01) (Table 1).

### 3.2. Tumour Size, Weight, and Progression

The non-treated positive control showed a higher median tumour size and weight compared to the group treated HSA (Group 2) (*p* < 0.05) (Table 1).

**Figure 1:**
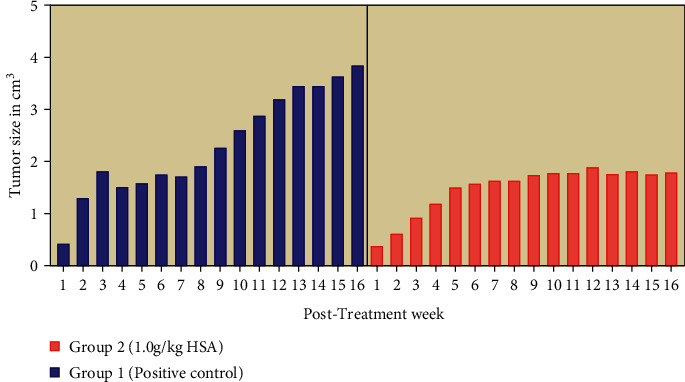
The progression of tumour size (cm^3^) in HSA-treated groups compared to the non-treated positive control. The data are presented as mean ± SEM. A mixed model two-way repeated measures ANOVA (*p* < 0.05 in all weeks). HSA, honey sugar analogues; positive control, group bearing breast cancer but received no treatment; TH, tualang honey; MH = manuka honey; HSA, honey sugars analogue; +ive control, group bearing breast cancer but received no treatment.

The tumour size measurements over the period of 16 weeks showed that the tumours in treated group (Group 2) had a slower size increment with a lesser median tumour size ≤1.90. The non-treated positive control group had a rapid progression over time reaching up to 3.84 cm^3^ in size. The statistical difference was not significant for the tumour size progression between the treated and control groups (*p* > 0.05). A significant difference was observed only in the last few weeks (*p* < 0.05) (Figure 1). HSA presented a slower tumour progression over period of 16 weeks (Figure 1).

### 3.3. Body Weights

**Figure 2:**
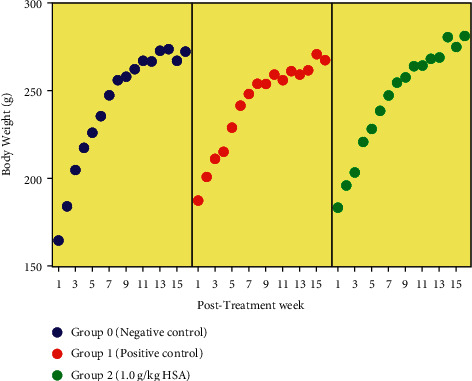
Body weight progression among all groups of rats. The data are presented as mean ± SEM and a mixed model two-way repeated measures ANOVA was conducted to analyze the results. A positive body weight progression was observed over time (*p* > 0.05). HSA, honey sugars analogue; negative control, normal rats; positive control, group bearing breast cancer but received no treatment; TH, tualang honey; MH, manuka honey; −ive control, normal rats; +ive control, group bearing breast cancer but received no treatment.

It was observed that body weights of the rats in all groups (treated and control groups) gradually increased over the experimental period of 16 weeks (Figure 2). No significant difference in the median body weights between all groups was observed at week 1 (*p* > 0.05). The rats in negative and positive control groups showed a higher median body weight compared to the HSA-treated group. At week 16, the median body weights of rats in controls and HSA treated group also presented no statistically significant difference (*p* > 0.05). However, the rats in negative and non-treated positive controls presented a higher median body weight compared to the treated group. The data for median body weights of rats in each group are presented in Table 2. The difference in percentage body weight change (BW change %) between all groups was found statistically not significant (*p* > 0.05). However, all the rats in the HSA-treated group showed a higher BW change % compared to the negative and non-treated positive controls (Table 2).

For further analysis on the weight gain, the actual body weights of rats were calculated at week 16 by subtracting the total tumour weight from the body weight of the rats obtained at week 16. The rats in the negative control group showed a higher median actual body weight compared to the HSA-treated group. However, the rats in the HSA-treated group presented a higher median actual body weight compared to the rats of the non-treated positive control. The rats treated with HSA also showed a significant higher percentage of change in actual body weight gain (ABW change %) than those in non-treated positive control. The difference in median actual body weight (ABW) of the treated group compared to the negative and positive control groups was not statistically significant (*p* > 0.05). Overall, treatment with HSA showed a positive effect on actual body weight gain compared to the non-treated positive control (Table 2).

### 3.4. Macroscopic Evaluation, Tumour Grading, and Histological Features

**Figure 3:**
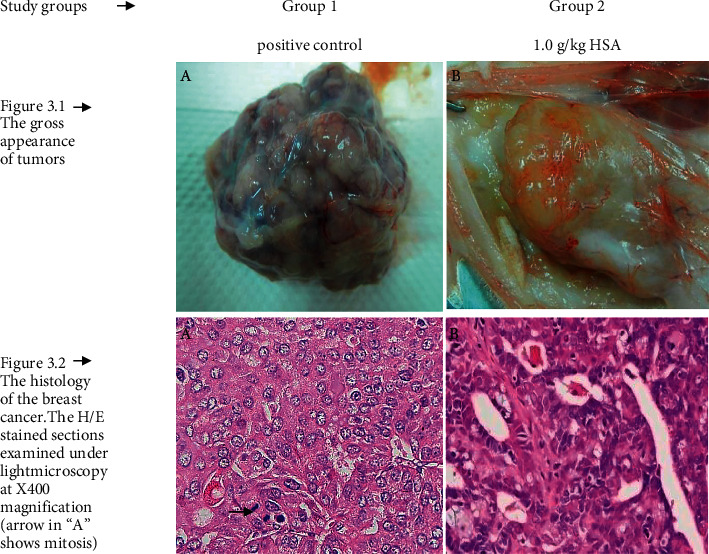
The gross morphology and histology of the breast tumours of rats in the HSA-treated group compared to the non-treated positive control. The majority of tumours in non-treated control group were of grade ІІІ with increased heterogeneous nuclei formation and mitotic activity (plate A arrow). A = +ive control (group bearing breast cancer but no honey treatment), B = 1.0 g/kg HSA. HSA, honey sugars analogue; positive control, group bearing breast cancer but received no treatment. (a) The gross appearance of tumors. (b) The histology of the breast cancer. The H/E stained sections examined under light microscopy at 400× magnification (arrow in “A” shows mitosis). TH, tualang honey; MH, manuka honey; +ive control, group bearing breast cancer but received no treatment.

Macroscopic evaluation of cancer masses showed that the HSA-treated group (Group 2) had tumours which were softer, paler, and smaller in size compared to those in the non-treated control. The tumour masses in the non-treated control (Group 1) were found to be larger in size, solid, and hard in consistency exhibiting areas of necrosis and hemorrhage. Some of these tumours exhibited pus-like material (necrotic tissue) exuding from the tumours when sectioned.  Figure 3 shows the effect of HSA treatment on gross appearance, size, and texture of tumours compared to the non-treated control.

**Figure 4:**
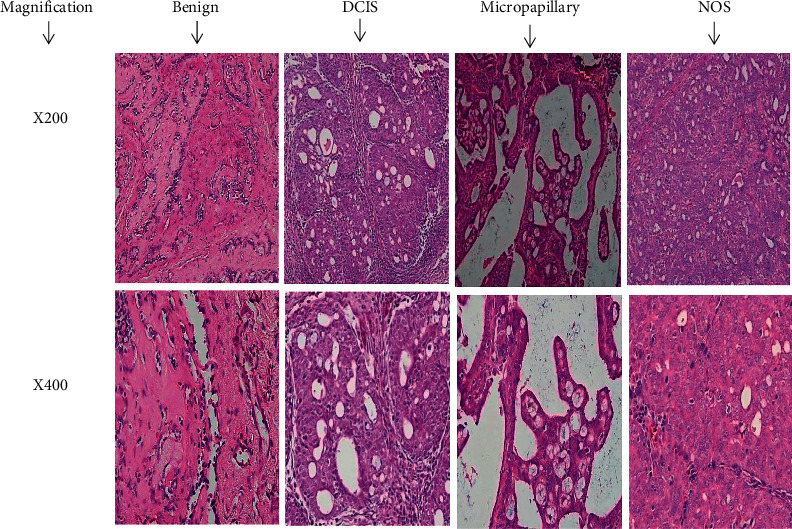
The histological patterns of tumors identified in breast cancer bearing rats among all groups. Cancers which developed in treated rats had less aggressive tumours behavior with more benign pattern compared to cancers developed in non-treated control; A = benign, B = DCIS, C = micropapillary, D = NOS. DCIS, ductal carcinoma in situ; NOS, not otherwise specified; DCIS, ductal carcinoma in situ; NOS, not otherwise specified.

The data of the histological grading for the cancer specimens are presented in Table 3. In all groups, the majority of the tumours were found to be adenocarcinomas. Tumours in the control group were observed to have increased heterogeneous nuclei formation which were hyperchromatic, vesicular, and highly pleomorphic, with moderate cytoplasm, and increased mitotic activity compared to the HSA-treated group which had fatty tissue with small nucleus and cystic spaces (Figure 3). Major types of carcinoma identified in both groups were Benign, DCIS (ductal carcinoma in situ), micropapillary, and NOS (not-otherwise specified) (Figure 4). The percentage of benign patterns was found higher in the HSA-treated group compared to the non-treated control (Table 4, Figure 4). Tumours grading results revealed that the majority of the tumours in the control group were of grade ІІІ compared to those in the group treated with HSA (Group 2) which exhibit tumours mainly of grade І and ІІ (Table 3, Figure 3).

### 3.5. Haematological Parameters

The effect of HSA on haematological parameters in breast bearing rats was determined. The results of haematological parameters of negative control were used to establish a normal or standard reference range. Treatment with HSA showed a potentiating effect on Hb, RBC, PCV, lymphocytes, and eosinophils compared to the non-treated positive control. While, HSA treatment showed a lowering effect on polymorphs, RDW, and monocytes. HSA showed a lower level of TWBC and a potentiating effect on platelets count. The detailed results for haematological parameters with statistical analyses are presented in Table 5.

### 3.6. Determination of Serum Level Concentration of Apaf-1, IFN-*γ*, TNF-*α*, and E2

**Figure 5:**
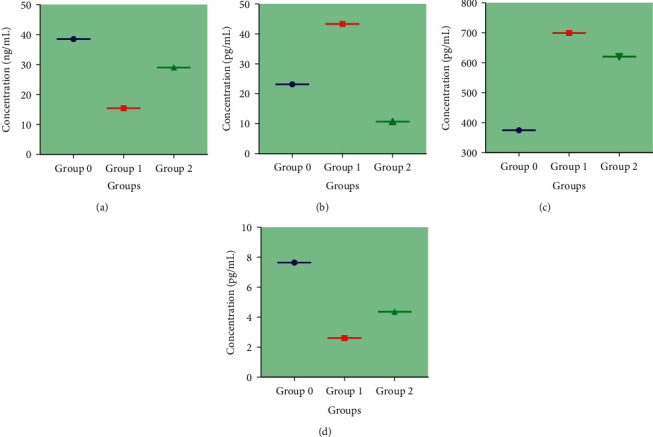
The serum level concentration of (a) Apaf-1 (ng/ml), (b) TNF-*α* (pg/ml), (c) E2 (pg/ml), and (d) IFN-(pg/ml) in the rats of the HSA-treated group compared to the rats of negative and positive controls. Group 0 = negative control (normal rats), Group 1 = positive control (group bearing breast cancer but received no treatment.), and Group 2 = 1.0 g/kg HSA. The data are expressed as median interquartile range (IqR) using Kruskal–Wallis test. Values are statistically significant, *p* < 0.05. Apaf-1, apoptotic protease activating factor 1; IFN-*γ*, interferon gamma; TNF-*α*, tumour necrosis factor alpha; E2, estradiol; HSA, honey sugars analogues; Apaf-1, apoptotic protease activating factor 1; IFN-*γ*, interferon gamma; TNF-*α*, tumour necrosis factor alpha; TH, tualang honey; MH, manuka honey.

Serum levels of Apaf-1, IFN-*γ*, TNF-*α*, and E2 in the negative control group (Group 0) were used to establish a normal reference range. HSA treatment showed a higher serum level median concentrations of proapoptotic proteins, Apaf-1 and IFN-*γ*, and a lower serum level median concentrations of antiapoptotic proteins, TNF-*α* and E2, compared to the non-treated positive control (Figure 5). A significant statistical difference was observed between all groups (*p* < 0.05).

### 3.7. Expression of Apaf-1, Caspase-9, p53, FASLG, FADD, and IFNGR1 in Tumour Specimens

**Figure 6:**
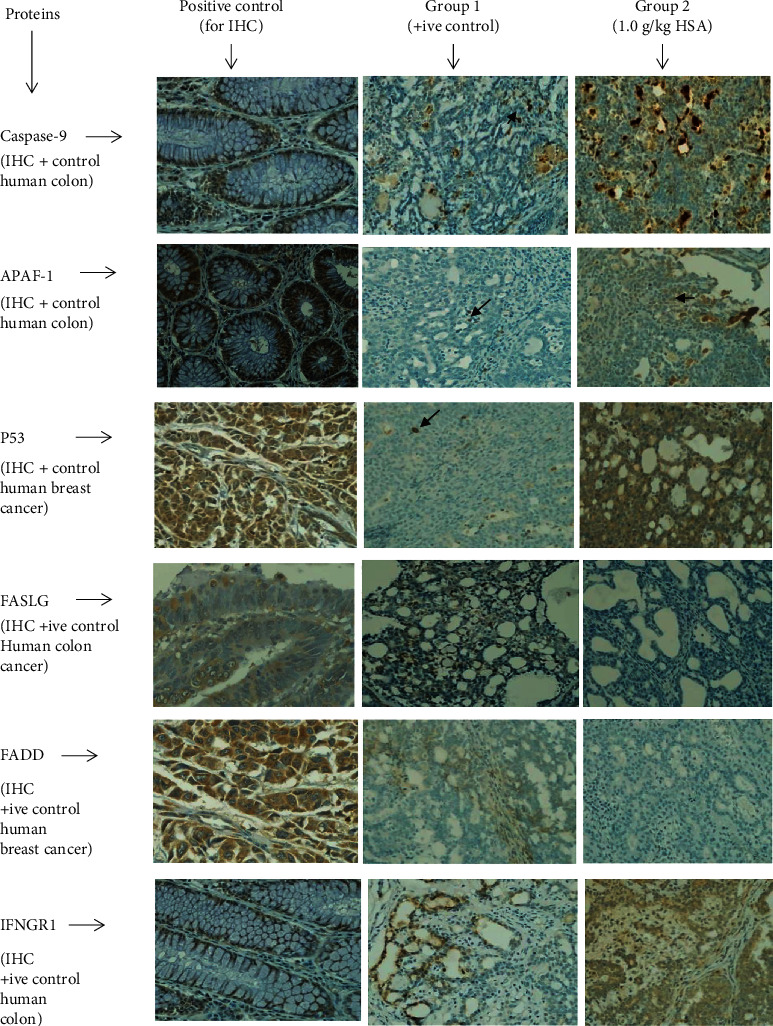
The immunohistochemical expression of proapoptotic proteins in HSA-treated tumors compared to the tumors of the non-treated control. A = positive control for immunohistochemistry (IHC) analysis, B = positive control for study (group bearing breast cancer but received no treatment), and C = 1.0 g/kg HSA. All specimens examined at 400× microscopic magnification and brown color show antibody positivity. FASLG and FADD showed no expression in tumours of all treatment groups, while, trumours in treated groups showed higher expression of proapoptotic proteins than those of the non-treated control. FASLG, fas ligand; FADD, fas-associated via death domain; IFNGR1, interferon gamma receptor 1; HSA, honey sugars analogue; FASLG, fas ligand; FADD, fas-associated via death domain; IFNGR1, interferon gamma receptor 1; TH, tualang honey; MH, manuka honey.

The cancer masses in HSA treated group showed a higher % expression of Apaf-1, caspase-9, p53, and IFNGR1 compared to those in the non-treated control (Table 6). Similarly, a higher percentage of immunopositive cells was also observed in HSA treated masses compared to those of the non-treated control (Figure 6). A significant statistical difference was observed between the two groups (*p* < 0.05).

Tumours treated with HSA showed no expression of FASLG and FADD (0% expression or positivity). A very minute expression of these proteins was observed in tumour specimens of non-treated positive control (Table 6 and Figure 6).

### 3.8. Expression of Bcl-xL, TNF-*α*, COX-2, and ESR1 in Tumour Specimens

**Figure 7:**
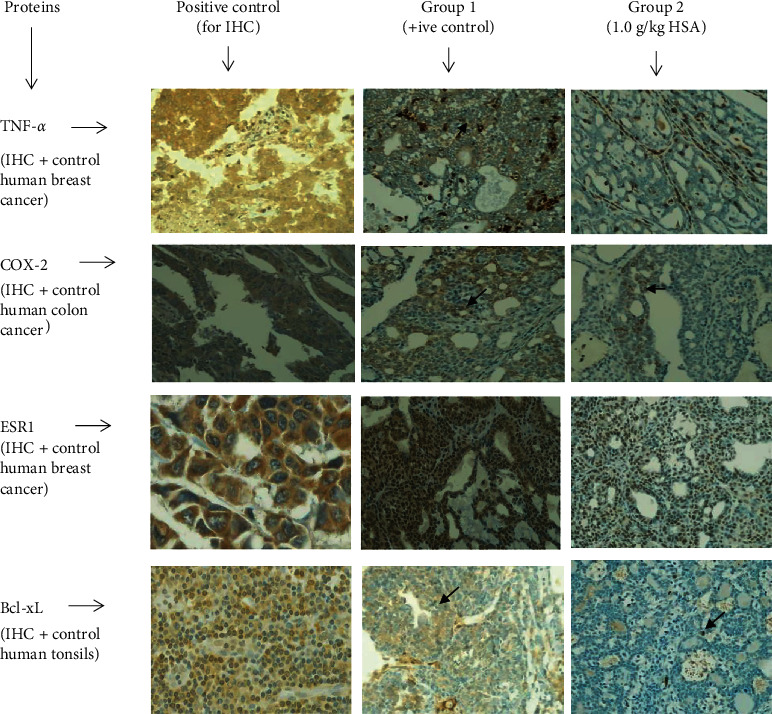
The immunohistochemical expression of antiapoptotic proteins in HSA-treated tumors compared to the tumors of non-treated control; A = positive control for IHC analysis, B = positive control for study (group bearing breast cancer but received no treatment), and C = 1.0 g/kg HSA. All specimens examined at 400× microscopic magnification and brown color show antibody positivity. TNF-*α*, tumour necrosis factor alpha; COX-2, cyclooxygenase 2; ESR1, estrogen receptor 1; Bcl-xL, B-cell lymphoma-extra-large; HSA, honey sugars analogue; TNF-*α*, tumour necrosis factor alpha; COX-2, cyclooxygenase 2; ESR1, estrogen receptor 1; Bcl-xL, B-cell lymphoma-extra-large; TH, tualang honey; MH, manuka honey.

Tumour specimens treated with HSA presented a significant lower % expression of Bcl-xL, TNF-*α*, COX2, and ESR1 and compared to the specimens in the non-treated control group (Table 6). A lower percentage of immunopositive cells for Bcl-xL, TNF-*α*, and COX-2 was also observed in treated tumours compared to those in the non-treated control. However, percentage of immunopositive cells for ESR1 in treated tumours was lower but not very significant compared to the tumours of control (Figure 7). A significant statistical difference was observed between these two groups (*p* < 0.05).

## 4. Discussion

Currently, honey has gained a renaissance as a complementary and alternative medicine. It seems to offer a real potential in providing novel usage or synergistic combinations to cure several ailments. It has been shown to exhibit antiproliferative [24], antitumoural [24], antineoplastic [25], antimutagenic [11], and anticancer [17] effects. The current study probes some novel findings of viable anticancer effects of honey sugars analogue (HSA) notching this ignored major profile of honey.

Our results showed that the oral administration of standard strength of HSA was noticeably effective to retard the size of primary tumours. The rats receiving these treatments also showed a decreased slower tumour progression (Figure 1), and lower tumour size, multiplicity, and weight compared to the non-treated positive control (Table 1). Such changes were also observed on gross macroscopic evaluation (Figure 3). Carcinogenesis is a multistep process. It can be divided into three main stages: initiation, promotion, and progression [26]. Cancer-therapeutic agents may act as antipromoting agents via intervening at initiation or promotion stages of carcinogenesis [26]. Thus, we can assume that HSA similar to honey may intervene at the initiation and or promotion stage to inhibit tumour progression, size, and weight. This is the reason that the treated tumours appeared smaller in our study. HSA may act as antimetastatic agent as observed by lower tumour multiplicity. Some of the breast lesions in our study were found to be completely vanished at the termination of the study. It has been reported that tumours can be diminished by chronic administration of low doses of cancer therapeutic drugs [27]. Thus, it can be hypothesized that HSA treatments behaved similarly. A study has shown that honey may modulate tumour multiplicity, size, weight, and progression [25]. That study investigated the cancer-preventive effects of honey using a different carcinogen DMBA (7,12-dimethylbenzanthracene). While, our study reports cancer-therapeutic effects of HSA using the carcinogen MNU which poses several advantages such as organ specificity and tumours of ductal origin compared to DMBA [19]. Thus, HSA may contribute to anticancer effects of honey.

Evaluation of detailed histological characteristics of the cancer masses has pivotal importance for the prognosis [28]. Our findings showed that the cancer masses in treated group were in grade I and II or less aggressive compared to the control which had majority of grade III (Table 3 and Figure 3). It was observed that the tumours in the control group were highly pleopmorphic with hyperchromatic nuclei, moderate cytoplasm arranged in sheets or nests, and acinar structures with increased mitotic counts. While, HSA treated tumours had low to moderate nuclear pleomorphism, fatty tissue with small lobules, and moderate cytoplasm and were likely to acquire benign patterns. The majority of the breast tumours in our study of MNU-induced model were invasive ductal carcinoma, and the commonest were of DCIS, micropapillary, and NOS. The tumours of benign type were found to be more frequently in treated group compared to the non-treated control (Table 4 and Figure 4). Thus, HSA seems to act at cellular level by reducing heterogeneous nuclei formation and mitotic activity to improve histological grading and patterns in cancer masses. This could probably lead to less aggressive types of tumours in treated group compared to the tumours of non-treated control with more aggressive patterns. Our previous such findings [17] with honey corroborate the effect of HSA.

Our study shows that HSA showed a slightly potentiating effect on body weight gain compared to the non-treated positive control (Table 2 and Figure 2). The positive control rats were not gaining as much weight as treated rats, which is perhaps due to cancer catabolism. Cancer is a catabolic state. Thus, cancer patients lose a lot of weight with worse outcomes [29]. It is hypothesized that HSA might be able to improve body weight gain. To rebut weight gain, one of the mechanisms explains that sugars in honey trigger a small spike in insulin levels, and insulin stimulates the release of tryptophan in the brain through insulin regulatory pathway. Tryptophan is converted to serotonin, which is then converted into melatonin at night. Melatonin in turn inhibits the release of insulin, thus further stabilizing blood sugar levels. This implication causes to down regulate the aerobic glycolytic pathway that is believed to play a vital role in lipogenesis, which may ultimately lead to an increase in body weight [30]. Thus, HSA in honey may play a vital role to gain body weight by triggering insulin pathway. Our previous findings [17] also confirm that honey attributes in body weight gain which authenticates the possible role of HSA in body weight amelioration.

Pre- and post-treatment studies have shown that breast cancer patients have deranged or abnormal blood parameters [31]. We investigated intriguing findings of blood parameters after the administration of HSA in the rats bearing breast cancer. HSA showed an increasing effect on the haematological parameters such as RBC, Hb, PCV, lymphocytes, and eosinophils. While, the treatment presented a slightly decreasing effect on the levels of RDW, polymorphs, and monocytes compared to the non-treated positive control (Table 5). Research has shown a lower level of RBC, Hb, MCV, MCH, MCHC, and lymphocytes in pre- and post-treatment breast cancer patients. Anaemia was also observed in these patients [31, 32]. A higher level of RDW, TWBC, and polymorphs has been reported in breast cancer patients [31]. Some studies have reported conflicting results on platelets count in pre- and post-treatment breast cancer patients [31, 32]. Our results suggest that HSA may alter or tend to normalize blood parameters on a slighter mode to abate breast carcinogenesis. Research has shown that exclusive honey feeding in the absence of any disease significantly modifies the haematological parameters [33]. Thus, we can presume HSA may ameliorate blood parameters in cancer patients similar to honey but at a slighter level than honey when compared to our previous finding on honey [17].

The understanding of the target mechanisms of action of natural products as cancer-therapeutic agents is essential to determine their applications in modern science. Additionally, these agents could represent a simple and promising strategy in the treatment of different types of human cancers. The uncontrolled proliferation and abnormal apoptosis leads to the occurrence and development of neoplastic cells with worst prognosis [34]. Some natural products such as honey can promote the apoptosis of cancer cells by ameliorating the expression of pro- and antiapoptotic proteins [17]. Therefore, it is worth a mention in this work that some events occurring related to changes in tumours progression and apoptosis caused by HSA, have been investigated. Our data reported that HSA caused to increase the expression of proapoptotic protein Apaf-1 at serum as well as at cancer tissues level (Figures 3 and 5). Similarly, these treatments were also found to potentiate the expression of other proapoptotic proteins, caspase-9 and p53 (Table 6, Figure 6). We may postulate that HSA caused to up-regulate the expression of Apaf-1, caspase-9, and p53 and thus may activate the intrinsic apoptotic pathway to promote apoptosis and antiproliferative effects. It is evidenced by regressed growth patterns and the low histological grading in the treated tumours. The possible mechanism demonstrates that HSA akin to chemotherapeutic agents may induce apoptosis through multiple signaling pathways that converge on the mitochondria to cause the release of cytochrome c. Cytochrome c binds to Apaf-1 in the presence of dATP/ATP (deoxyadenosine triphosphate/adenosine triphosphate), which then binds to procaspase-9 to form a cytochrome c-Apaf-1-caspase-9 complex, called apoptosome. Apoptosome enables enzymatic self-activation of caspase-9 that subsequently activates procaspase-3. This ultimately results in cell death [35]. Similar pathway has been reported in apoptosis via honey [17] based on our previous findings. Honey mediates apoptosis mainly through the intrinsic apoptotic pathway and by enhancing proapoptotic proteins expression such as p53 and caspase-9 [36–38]. We can assume that HSA may act as therapeutic agents against breast cancer by modulating the expression of Apaf-1 and caspase-9 with involvement of p53. Our results showed no evidence of the expression of FASLG and FADD, hence no involvement of caspase-8 or the extrinsic apoptotic pathway in HSA mediated apoptosis, similar to our previous findings on honey (Table 6, Figure). Our results are in line with another study which demonstrated that honey induces intrinsic or caspase-9 apoptotic pathway in breast cancer with no evident involvement of caspase-8 pathway [24].

The concomitant decrease of TNF-*α*, COX-2, and Bcl-xL was observed in the treated tumours compared to those of control (Table 6, Figure 7). It suggests that administration of HSA can also lead to lower tumour cells proliferation and increased apoptosis by reducing the expression these antiapoptotic factors and or proteins such as TNF-*α*, COX-2, and Bcl-xL. Research has shown that a higher level of TNF-*α*, COX-2, and Bcl-xL implies that tumours have a rapid growth rate, and in some cases, indicates severe aggressiveness, metastatic behavior, and poor prognosis [39, 40]. Therefore, the reduction of TNF-*α*, COX-2, and Bcl-xL observed in our study might indicate that the treatment with HSA could modulate tumour growth and progression. TNF-*γ* is produced by monocytes [41], and our study also shows that HSA treatments cause to lower the monocytes level in blood. This validates that HSA hinders this signaling pathway by lowering TNF-*γ* as well as monocytes to cause the anticancer effects. It can also be hypothesized that COX-2 inhibition by HSA may cause lower tumour multiplicity through reduction in inflammation caused by COX-2. The proposed mechanism by which HSA may inhibit COX-2-induced inflammation in carcinogenesis is supported by previous findings of honey [17, 42]. These findings proposed that similar to honey, HSA may inhibit inflammation through suppression of the NF-*κ*B pathway by blocking this signaling pathway. This pathway activates the IKK (I*κ*B kinase) complex through interfering with the phosphorylation, ubiquitination, and degradation of I*κ*B (inhibitor of kappa B) proteins. This will then prevent the translocation of NF-*κ*B dimers (p65 and p50) into the nucleus and finally resulting in the reduction of COX-2, TNF-*α*, iNOS, IL-6 (interleukin-6), PGE2 (prostaglandin E2), and NO production [42]. Thus, it may be presumed that HSA may intervene in these inflammatory signaling pathways similar to honey to downregulate the COX-2 as well as TNF-*γ* expression. COX-2 expression is regulated by TNF-*α*, and our study also shows a lower expression of TNF-*α*. This further validates the hindering of this signaling pathway by HSA.

Investigations on human cancer cells have shown that the reduced expression of immune-regulatory factors such as IFN-*γ* and IFNGR1 is tightly associated with poor prognosis and more aggressive behavior of cancer [43]. The higher expression of IFN-*γ* and IFNGR in HSA-treated rats as observed in our study may modulate breast carcinogenesis. The results of our study are supported by a research demonstrating that honey may regulate immune markers such as IFN-*γ* to inhibit tumour formation [44], supporting possible effect of HSA. The possible mechanism explains that binding of IFN-*γ* to IFNGR1 initiates autophosphorylation and transphosphorylation with the activation of the Janus-activated kinases (JAKs). This in turn phosphorylates the intracellular domain of IFNGR1 leading to the recruitment of Stat1 proteins [43]. The phosphorylated Stat1 proteins form reciprocal homodimers and dissociate from the receptor complex. It translocates at the nucleus and regulate the transcription of IFN-*γ*-inducible genes to induce apoptotic and immunoregulatory effects [43]. Our study provides evidence that HSA may act as immune potentiators by inducing IFN-*γ* and IFNGR1 expression, similar to honey as reported previously [17].

The results obtained in our study demonstrate that HSA showed a lowering effect on E2 concentration at serum level (Figure 5), and ESR1 at cancer tissues level (Table 6 and Figure 7). Breast cancer patients had a higher level of E2 and ESR1 mediated activity [45], which promotes cell proliferation and suppresses apoptosis by directly modulating the genes transcription. Thus, estrogen is considered as an important target in breast cancer treatment. Treatment with estrogen-lowering drugs shrinks breast tumours [46]. Thus, HSA may act as estrogen-lowering drugs and shrink tumours size as observed in our study. The inhibition of ESR1 may decrease the risk for hormonal breast cancer [45]. In mechanism, estrogen receptors (ERs) bind to estrogens to dimerize and then translocate into the nuclei. These complexes then bind to the specific DNA base sequences called estrogen-response elements (EREs) resulting in transcription and translation of the estrogenic effect in the targeted tissue [47]. This signaling cascade induced by estrogens may be modulated at any stage [47]. Thus, HSA may possibly modulate E2 and ESR1 and hinder this signaling pathway to suppress tumours growth. Administration of exogenous or synthetic estradiol (E2) is used as a treatment in ER positive breast cancer to cope ER proliferative pathway [47]. It is quite possible that honey, recognized as a natural phytoestrogen [6], plays its role in modulating the endogenous estrogen and estrogen receptors, stimulating the apoptotic pathway, and possibly HSA may play a role in this pathway as a constituent of honey. Research has shown that honey exhibits estrogen agonistic effect at higher concentrations tested such as 20–100 *μ*g/mL and antagonistic effect at lower concentrations such as 0.2–5 *μ*g/mL in *in vitro* model [48]. This antiestrogenic effect of honey is attributed to its flavonoids or polyphenols content [48]; now, our findings recommend that HSA may also play a role in this phenomenon.

Equally important to this finding is the fact that honey sugars analogue or HSA exhibits similar effects as honey concentrations tested with some efficacy variations, supported by our previous findings on honey [17]. However, on comparing with previous studies [17], it is observed that HSA did not show better effects in terms of some haematological, serological, and cancer tissue level parameters. This is perhaps due to the fact that honey is a mixture of several health boosting components compared to simple sugars or HSA.

## 5. Conclusion

HSA treatment results in modulation of body weight, haematological and serological parameters, and cancer antiproliferative activity through amelioration of expression of pro- and antiapoptotic proteins. Thus, HSA may well act as antibreast cancer agents as a major constituent of honey via multiple protective mechanisms. Certainly, a number of complex mechanisms may be involved in tumours modulatory effects of HSA, similar to honey. Thus, it is vindicated that HSA behaves akin to honey and may play a role in overall efficacy of honey. Our findings suggest further work to investigate more about HSA for studying more parameters at molecular level such as antiproliferative effects via osmotic potential, antitoxicity effects, and apoptotic genes expression using advanced techniques in *in vitro* and *in vivo* models.

## Figures and Tables

**Table 1:** Tumour multiplicity, % reduction, size, and weight in HSA-treated group compared to the non-treated control.

Groups			
Tumor	1 Positive control	2 (1.0 g/kg HSA)	*p* value
^*∗*^Multiplicity	6 (4.1)	3 (3.5)	0.462
^*∗*^% Reduction	2 (0)	58.53 (37.97)	≤0.01
^*∗*^Size (cm^3^)	1.41 (2.21)	0.23 (0.54)	≤0.01
^*∗*^Weight (g)	2.15 (2.36)	1.25 (2.53)	0.011

^*∗*^Kruskal–Wallis test. The data are expressed as median interquartile range (IqR). Values are statistically significant when *p* ≤ 0.05. Multiplicity, no. of tumours developed; % reduction, the percentage reduction in size of primary tumours; HSA, honey sugars analogue; positive control, group bearing breast cancer but with no treatment.

**Table 2:** Body weight measurements of rats among all groups at week 1 and week 16.

Groups				
Body weight	0 Negative control	1 Positive control	2 (1.0 g/kg HSA)	*p* value
BW at week 1	168.1 (29.15)	192.7 (91.4)	126 (29)	0.300
BW at week 16	268 (12.33)	271.6 (29)	269.2 (43.25)	0.392
BW change (%)	65.44 (29.35)	33.24 (91.05)	63.82 (99.86)	0.182
ABW at week 16	273 (31.25)	243.29 (19.10)	271.565 (52.98)	0.07
ABW change (%)	65.44 (39.36)	28.42 (69.47)	45.195 (99.1)	0.110

^a^Kruskal–Wallis test. Data are expressed as median interquartile range (IqR). Values are statistically significant when *p* ≤ 0.05. BW, body weight; ABW, actual body weight; HSA, honey sugars analogue; negative control, normal rats; positive control, group bearing breast cancer but received no treatment.

**Table 3:** Grading of tumours in groups treated with HSA compared to the tumours of non-treated control.

Groups		
Tumor	1 positive control	4 (1.0 g/kg HSA)
Total no.	39	26
^*∗*^Grade І (%)	5 (22.16)	11 (42.3)
^*∗*^Grade ІІ (%)	17 (21.41)	11 (42.3)
^*∗*^Grade ІІІ (%)	23 (50.31)	4 (15.38)

^*∗*^Fisher exact test: statistically a significant difference between the groups, *p* < 0.05.

**Table 4:** The % age of histological patterns identified in the HSA-treated group versus non-treated positive control.

No. of tumors (amount %)					
Group	Total	Benign	DCIS	Micropapillary	NOS
1 positive control	44	1 (4.07)	2 (7.11)	3 (7.15)	38 (81.62)
4 (1.0 g/kg HSA)	27	4 (7.13)	1 (3.56)	2 (10.70)	20 (78.56)

HSA, honey sugars analogue; DCIS, ductal carcinoma in situ; NOS, not otherwise specified; positive control, group bearing breast cancer but received no treatment.

**Table 5:** The haematological parameters of the HSA-treated group compared to the control.

Groups				
	1 Negative control	2 positive control	3 (1.0 g/kg HSA)	*p* value^a^
RBC (10^12^/L)	8.34 (0.49)	5.1 (1.8)	6.84 (1.0)	0.002
Hb (g/dl)	16.1 (0.73)	10.34 (2.41)	15.0 (1.44)	0.002
PCV (%)	45 (3.24)	34 (7.24)	44 (7.4)	0.008
MCV (fl)	68.4 (3.24)	64 (1.74)	64 (4.4)	0.012
MCH (pg)	20.4 (1.5)	20.59 (2)	20 (1.4)	0.168
MCHC (g/L)	31 (1.5)	31.4 (2.14)	31 (4)	0.061
RDW (%)	13.8 (1.16)	14.04 (1.71)	12.12 (1.7)	0.01
TWBC (10^9^/L)	5.04 (1.34)	7.3 (7.51)	5.5 (3.14)	0.02
Polymorphs (%)	31 (9.04)	44.4 (13)	31 (15)	0.01
Lymphocytes (%)	69 (7.1)	47 (16.24)	67 (14.4)	0.013
Monocytes (%)	2.5 (2.4)	2.4 (2.4)	1 (2.4)	0.230
Eosinophils (%)	0 (1.5)	0 (1.84)	1 (1)	0.101
Basophils (%)	0	0	0	1
Platelets (10^9^/L)	828 (201.14)	617.4 (106.04)	767 (254)	0.01

^a^Kruskal–Wallis test. The data are expressed as median interquartile range (IqR). Values are statistically significant when *p* ≤ 0.05. FBC, full blood count; RBC, red blood cells; Hb, haemoglobin; PCV, packed cell volume; MCV, mean corpuscular volume; MCH, mean corpuscular haemoglobin; MCHC, mean corpuscular haemoglobin concentration; RDW, red cell distribution width; HSA, honey sugars analogue; negative control, normal rats; positive control, group bearing breast cancer but received no treatment.

**Table 6:** The immunohistochemical expression of pro- and antiapoptotic proteins in tumours treated with HSA compared to the tumours of the non-treated control.

Groups		
Tumors	1 positive control	2 (1.0 g/kg HSA)
Total no.	39	26
No. of caspase-9-positive tumors (% expression)	13 (31)	19 (73)
No. of Apaf-1-positive tumors (% expression)	16 (27.6)	16 (61)
No. of p53-positive tumors (% expression)	15 (32.6)	18 (69)
No. of FASLG-positive tumors (% expression)	16 (37.6)	20 (77)
No. of FADD-positive tumors (% expression)	14 (22.6)	0
No. of IFNGR1-positive tumors (% expression)	21 (11)	0
No. of TNF-*α*-positive tumors (% expression)	31 (86)	19 (73)
No. of COX-2-positive tumors (% expression)	27 (56)	14 (53)
No. of ESR1-positive tumors (% expression)	33 (71)	18 (69)
No. of Bcl-xL-positive tumors (% expression)	32 (67.6)	14 (53)

Kruskal–Wallis test; statistically significant differences between the groups, *p* < 0.05. FASLG, fas ligand; FADD, fas-associated via death domain; IFNGR1, interferon gamma receptor 1; TNF-*α*, tumour necrosis factor alpha; COX-2, cyclooxygenase- 2; ESR1, estrogen receptor 1, Bcl-xL, B-cell lymphoma-extra-large; positive control, group bearing breast cancer but received no treatment; HSA, honey sugars analogue.

## Data Availability

The data sets generated during and/or analyzed for this study project have been included in the main text. The data pertaining to ethics and or any other supplementary materials or any required data are available from the corresponding authors upon reasonable request.
